# A Novel Hybrid Intelligent Indoor Location Method for Mobile Devices by Zones Using Wi-Fi Signals

**DOI:** 10.3390/s151229791

**Published:** 2015-12-02

**Authors:** Manuel Castañón–Puga, Abby Stephanie Salazar, Leocundo Aguilar, Carelia Gaxiola-Pacheco, Guillermo Licea

**Affiliations:** Facultad de Ciencias Químicas e Ingeniería, Universidad Autónoma de Baja California, Calzada Universidad 14418, Tijuana 22390, Mexico; abby.salazar@uabc.edu.mx (A.S.S.); laguilar@uabc.edu.mx (L.A.); cgaxiola@uabc.edu.mx (C.G.-P.); glicea@uabc.edu.mx (G.L.)

**Keywords:** wireless networks, indoor location, location-based services, wireless/mobile applications, Type-2 fuzzy inference system, data mining

## Abstract

The increasing use of mobile devices in indoor spaces brings challenges to location methods. This work presents a hybrid intelligent method based on data mining and Type-2 fuzzy logic to locate mobile devices in an indoor space by zones using Wi-Fi signals from selected access points (APs). This approach takes advantage of wireless local area networks (WLANs) over other types of architectures and implements the complete method in a mobile application using the developed tools. Besides, the proposed approach is validated by experimental data obtained from case studies and the cross-validation technique. For the purpose of generating the fuzzy rules that conform to the Takagi–Sugeno fuzzy system structure, a semi-supervised data mining technique called subtractive clustering is used. This algorithm finds centers of clusters from the radius map given by the collected signals from APs. Measurements of Wi-Fi signals can be noisy due to several factors mentioned in this work, so this method proposed the use of Type-2 fuzzy logic for modeling and dealing with such uncertain information.

## 1. Introduction

In spite of computer devices being frequently used in changing environments, they still do not adapt well to these changes. Devices with situational awareness should understand and handle the context in order to execute the correct task at the proper moment without, or with minimal, human intervention. Context is defined as every piece of information that can be used to characterize the situation of an entity; at the same time, an entity can be a person, place or object that is considered relevant to the interaction between the user and one application, including the user and the application themselves [1]. Depending on the particular activity in which the relevant information is involved, in certain cases, it is hard to obtain several parameters that define the context, for example the mood of the user.

Several authors consider the location of the user as an important parameter to know the context of a situation [2]. In addition, the use of mobile devices is increasing, and the tendency is the development of technology that brings increasingly more embedded resources in it that provide more data and diversity of information from the surrounding environment [3]. This fact enables devices to obtain and use the information as an implicit input that positively affects the behavior of applications [1]. Location-based systems are more frequent, and considering that most people spend the majority of their time inside walls [4], there is an interest for researchers and developers about the estimation of indoor location.

Different types of methods and techniques have been developed to estimate indoor location using different technologies, but there is no standard to do it yet. Conventional location techniques use the Global Positioning System (GPS), Bluetooth or radio frequency identification (RFID), among others technologies [5]. Nevertheless, a disadvantage of GPS is that satellite signals are blocked by obstacles, such as walls, besides that weather variations or the presence of buildings result in approximations with error of meters, so it is not possible to use this system as a method for indoor location [6,7]. On the other hand, Bluetooth technology has a limited coverage, and it is designed for too short communication distances to solve the indoor location problem. Finally, radio frequency is an expensive solution because it implies the installation of many sensors in the space to perform the location [7]; therefore, it is not an economically-viable method. Consequently, alternative technologies are required that satisfy a particular grade of adaptability to established infrastructures that accomplish, with the indoor location function, ease of use and an accessible cost, such as the use of Wi-Fi-based technologies [5].

**Figure 1:**
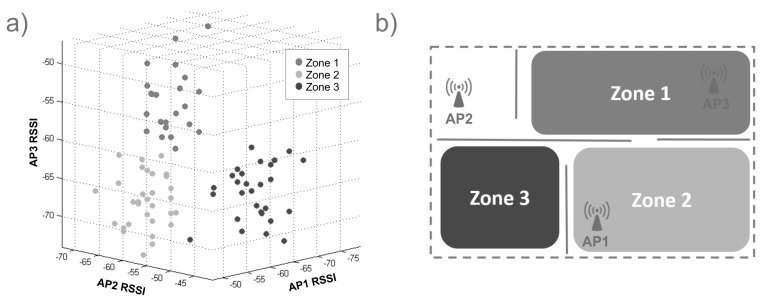
(**a**) 3D radio-map plot of RSSI data collected; (**b**) an example map of three zones and three access points distribution.

Indoor location methods (ILM) using wireless local area networks (WLAN) have recently become popular because WLANs give almost total indoor coverage [8]. These methods commonly use a scene analysis technique [9] based on fingerprinting. The proposed method fingerprints the RSSI (received strength signal indicator) values from at least three APs to avoid mirror faulty samples that correspond to more than one zone and to be able to position the device in the zone. The collection of fingerprints constructs radio maps of the area. The approach depends on the way the fingerprints are collected over an area, and some authors divide the scene into cells, as [9,10]. The present approach divides the area into zones of interest. When obtaining a set of data of RSSI from Wi-Fi networks from a determined infrastructure, the different combinations of them can form a group of zones that help to locate a device. Figure 1a shows an ideal 3D plot example of RSSI data collected from three different APs; gray points are from Zone 1, light gray points from Zone 2 and dark gray points from Zone 3. In Figure 1b, an example map shows three different zones and three APs to collect the data in Figure 1a.

Despite the acceptable coverage that WLANs offer, there are challenges using Wi-Fi to estimate location, primarily due to variations and noise on the RSSIs. The signals’ availability, traffic, propagation effects, changing or removing the APs’s original positions, moving objects or people, structural changes and other devices in the same band (as Bluetooth and ZigBee) can be interference sources for ILMs [11]. RSS is not a normal distribution in indoor environments, causing deterministic methods that only use the average feature to not have good accuracy [12]. The work in [8] has a method that detects and mitigates faulty measurements; it computes and replaces the faults with a more reliable metric. This work managed RSSI selection from a performance improvement point of view by removing certain unstable AP measurements. Secondly, ILMs using Wi-Fi are divided into two phases or stages, traditionally called offline (also calibration, pre-processing or sampling) and online (also post-processing or location). Challenges of fingerprinting for ILM using Wi-Fi arise mainly because of time and processing costs during the offline phase. The conventional methods to avoid these problems uses radio propagation, but fail to capture structure (dimensions) nor dynamic (people, elevators moving) details. RSS radio maps (collection of fingerprints) at different times or from different devices use limited calibration, because the results may vary with changes in these conditions. The majority of the authors uses the probabilistic k-nearest neighbor (kNN) algorithm that uses the probability of RSS as a weighting rather than an average value of the simple RSS to handle these problems on fingerprinting; see [4,9,13,14]. Furthermore, a differential radio map using the difference between the RSSIs from each AP, instead of the raw fingerprint, makes it adaptable to the dynamic indoor environment and different mobile devices by modeling a common mode noise; see further information in [15].

In previous work, we started exploring with experiments that consisted of collecting RSSI data and embedding the method in a developed wristband device using a Type-1 fuzzy logic system (FLS) [16]. The next approaches included the first tests of a developed tool for Android mobile devices to collect the Wi-Fi data, applying the data mining fuzzy C-means technique, constructing a Mamdani FLS and using the generated system as the locator [17]. Finally, in [18], we implemented subtractive clustering and Takagi–Sugeno FLS in the developed tool with better data mining results, but with location faults in closer zones.

In this paper, the proposed location method pre-processes the collected data to mitigate faulty measurements and is a hybrid intelligent approach consisting of data mining and fuzzy logic systems (FLS). The data mining technique used is subtractive clustering, which is a semi-supervised algorithm that estimates the number of groups and the center of each group of a set of data [19]. Clustering of numerical data is part of the basis of different algorithms and classification modeling systems with the purpose of obtaining data groups from a significant set of data to get a representation of the system behavior [20,21]. Correspondingly, the obtained cluster centers from an RSSI radio map are used to generate fuzzy rules that describe the indoor location Type-2 FLS. Using Type 2 permits modeling uncertainties of the RSSIs. Once the Takagi–Sugeno FLS is generated, it is ready to be evaluated with new inputs to estimate the location.

## 2. Experimental Section

**Figure 2:**
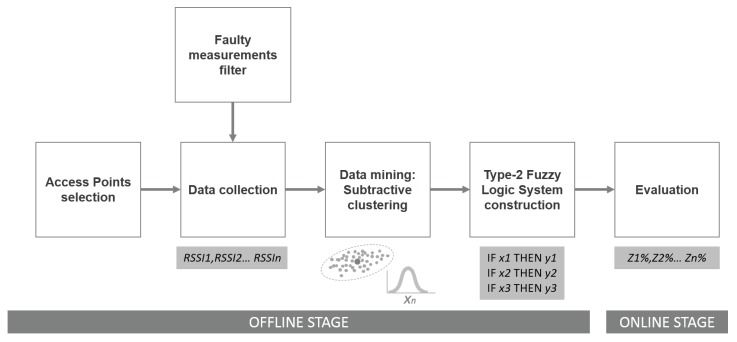
Block diagram of the proposed indoor location method (ILM).

This work presents an alternative method to estimate the location of devices in indoor spaces using a radio map of RSSIs of at least three APs. Once the radio map is obtained, the data mining algorithm, called subtractive clustering, tries to find clusters of the dataset after being given an influence radius parameter by an expert; see the details in Section 2.3. Numerical data from the found clusters help to construct Gaussian membership functions and fuzzy rules that conform to a fuzzy inference system that maps relations between inputs (RSSI vector) and outputs (membership of zones vector). Figure 2 shows the simplified process divided into two stages, online and offline (mentioned in Section 1). The most expensive stage is offline, which includes the APs’ configuration, the collection of RSSIs data, pre-processing of the collected data and usage of data mining methods to find clusters in the collected data in order to construct the Type-2 FLS. Meanwhile, the online stage only consists of the use of the FLS for indoor location and possible applications that make use of it.

### 2.1. Access Point Selection

This method uses a supervised access point selection. An expert defines the most viable APs to use in order to be able to estimate the position of a mobile node with at least three RSSI values used as coordinates to avoid mirror sets of data. At least three APs should have enough coverage in every zone for location estimation. Experimentation with the kNN-based algorithm for indoor location demonstrates that location accuracy will not increase when the number of APs increases to 4, because of inaccurate APs for certain test points [10].

**Figure 3:**
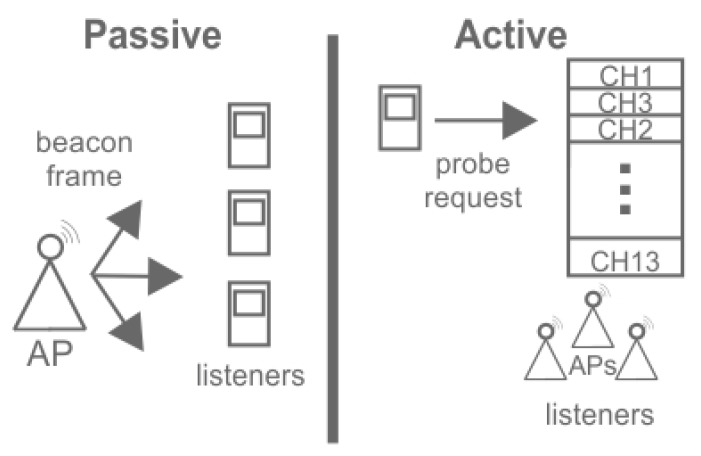
Passive and active scenarios.

On the other hand, passive and active indoor scenarios using APs can be used. The difference between a passive and active scenario is explained using Figure 3. Our proposed method uses a passive scenario where the mobile devices listen to the beacon frame that the APs continually broadcast through the channels with the AP information. Meanwhile, other methods implement an active scenario where mobile devices send a probe request over the different channels in the band asking for the APs’ (listeners) response for information (Service Set Identifier, Received Strength Signal, Media Access Control Address). Some scenarios configure a determined number of APs as listeners in monitor mode to capture the probe or beacon request that only devices with location purposes execute [8]. The disadvantages of this kind of scenario is that access points in monitor mode should be adapted and that ordinary functions are limited.

As mentioned in the Introduction, RSSIs can be missed because of problems in the selected APs. Algorithm 1, shown below, was developed to remove missing signals during the collection of data to mitigate faulty measurements in the next steps.

**Algorithm 1** Algorithm to remove missing RSSI during the online stage.**Require:** SSIDs: list of selected APs names; RSSIs: empty list of storage values; error = false; maxErrors for maximum errors; and errorCounter = 0.1:**while** enters the first time and do while error equals true **and** errorCounter < maxErrors **do**2:  scan the area to check broadcast of available APs3:  obtain a list Devices with information of every available AP found4:  **for** *i*=0 to Devices size **do**5:      **for** *j* to SSIDs size **do**6:            **if** Devices *i* element equals SSIDs *j* element **then**7:                    save RSSI from Devices *i* element on RSSIs *j* element8:            **end** **if**9:      **end** **for**10:  **end** **for**11:  **if** RSSIs have at least three not-empty elements **then**12:      error is false13:  **else**14:      error is true, errorCounter + 1 and empty RSSIs15:  **end** **if**16:**end** **while**17:**if** errorCounter >= maxErrors **then**18:  **print** error message and empty RSSIs19:**end** **if**20:**return** RSSIs

### 2.2. Data Collection and Fingerprinting

Important points of fingerprinting of Wi-Fi signals for this case study are listed below:

- Areas where the device is to be located should be defined. Several results obtained during this experimentation indicate that zones with a distance of about less than 5 m, approximately, should use 4 APs for collection to have better accuracy; zones with a greater distance can use 3 APs.
- A software tool for Android mobile devices was developed to collect the Wi-Fi signals and to estimate location.
- RSSI from at least three APs for each zone should be collected in each zone covering every space; each zone can be better described with more diversity and a greater quantity of data.
- This approach enables one to avoid collection of data in one zone that was between two zones; the outputs of the fuzzy inference system (FIS) in this case can indicate a membership grade of 50 and 50 in the zones between which it was located.

Table 1 shows a sample of collected data. In this example, each input corresponds to each signal from three APs. At the same time, three different outputs were generated (online during the collection) with a value of 1 corresponding to the zone where the value was taken.

**Table 1:** Sample of collected data for Scenario 1.

Zone	Input 1	Input 2	Input 3	Output 1	Output 2	Output 3
1	−59	−74	−51	1	0	0
1	−58	−55	−71	1	0	0
1	−56	−70	−49	1	0	0
2	−62	−75	−60	0	1	0
2	−57	−84	−54	0	1	0
2	−61	−80	−59	0	1	0
3	−75	−64	−95	0	0	1
3	−64	−54	−51	0	0	1
3	−74	−65	−52	0	0	1
…	…	…	…	…	…	…

#### 2.2.1. Materials

The Wi-Fi device to sense the RSSIs from each AP and where the tests were executed was a Samsung Galaxy Tab 4 7.0, 1.4-Hz Quad Core Processor with a 4-KmA-hour battery. The four routers or APs used were Belkin Wireless G Router 2.4 GHz 802.11g Model No. F5D7230-40 (AP1), Cisco-Linksys Model WAP54G Wireless-G 802.11g access point (AP2), one D-Link Wireless Router 2.4 GHz 802.11g Model DI-524 (AP3) and one Air Port Extreme by Apple Model No. A1034 (AP4).

#### 2.2.2. Software

**Figure 4:**
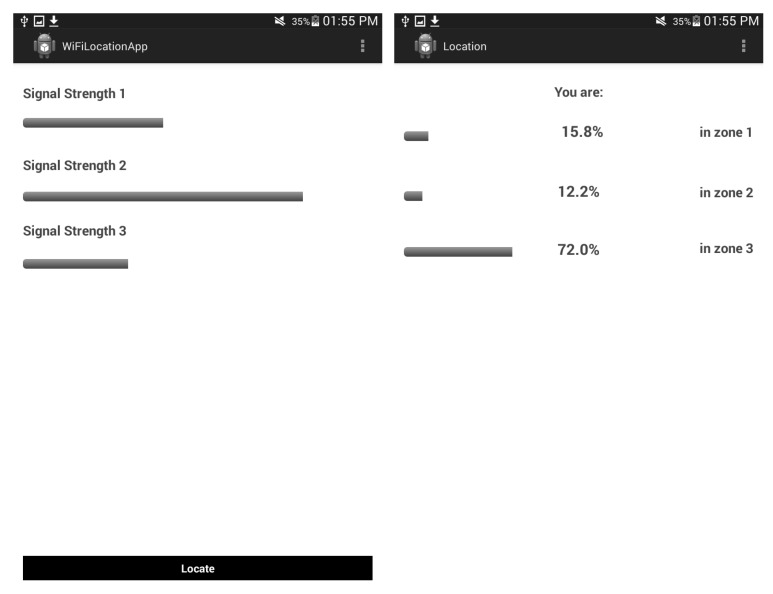
Demo of the Android application for indoor location.

Figure 4 shows images of the Android application developed that implements the ILM proposed in this work. Offline and online stages were executed in the mobile device with this application. The Android application uses the JT2FIS Java Class Library [22] to incorporate the data mining and fuzzy tools.

#### 2.2.3. Case Study

**Figure 5:**
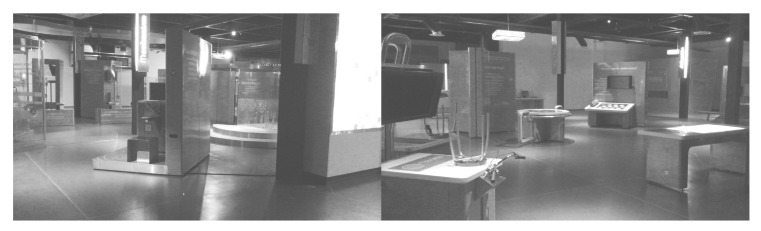
Interactive museum, ILM scenario.

**Figure 6:**
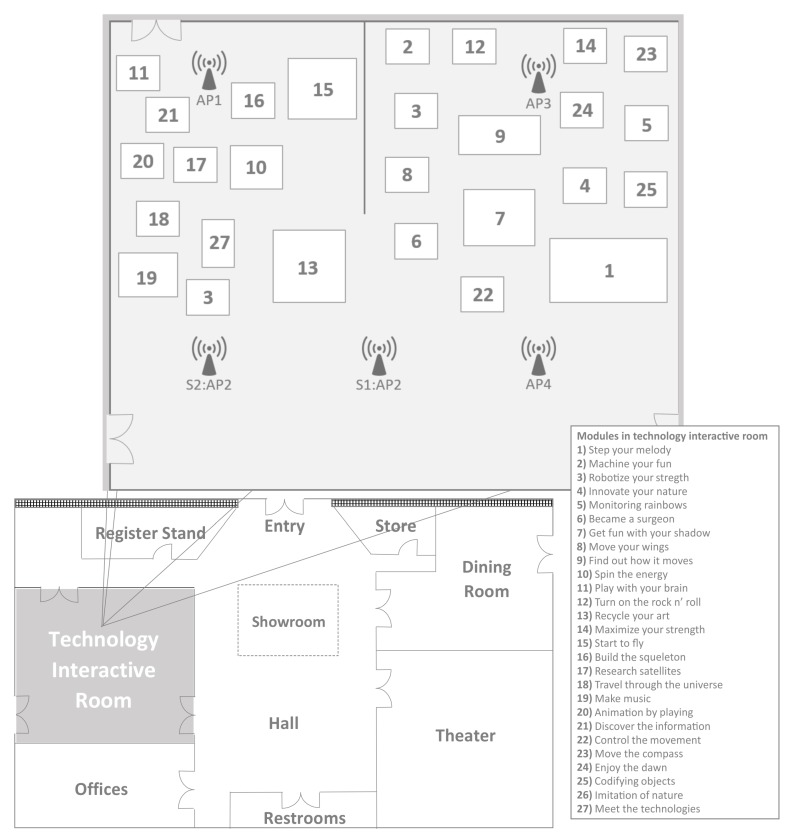
The museum’s 1st floor for the Indoor location methods (ILM) scenario.

This case study proposes the indoor location inside an interactive museum with a device with the Android system using wireless signals with at least three different established access points in the area. The museum has three active floors with different interactive rooms on each floor. The obtained data were taken from the Technology Interactive Room on the first floor; Figure 5 shows the ILM scenario. This room has 27 interactive modules about science and technology; the details are in Figure 6. The case study was divided into two scenarios: one with 3 APs and 3 zones that were not very close to each other; and the other with 4 APs and 3 closer zones. The position of AP2 was modified for both scenarios. The AP2 position is indicated with Sn : in Figure 6, as well. For experimental purposes, in Scenario 1, Modules 3, 6 and 7 represents Zone 1, Zone 2 and Zone 3, respectively. Only Scenario 2 adds a fourth access point and the proposed Algorithm 1 to mitigate faulty APs measurements. Modules 10, 16 and 17 represents Zone 1, Zone 2 and Zone 3, respectively. The goal of Scenario 2 was to achieve better accuracy after obtaining bad results in previous work with closer zones.

**Figure 7:**
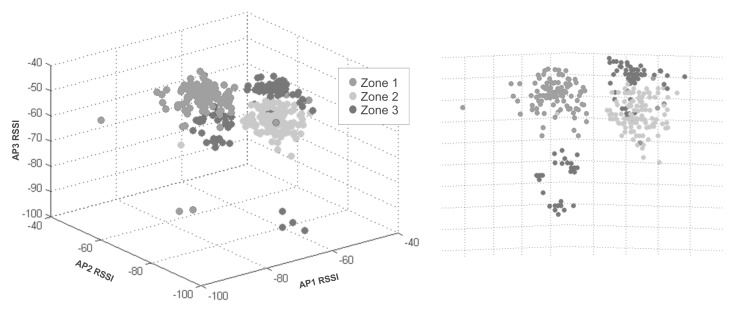
Plot of collected data from Scenario 1.

Because in Scenario 1, 3 APs were used, it is possible to plot the data in a 3-axis plot; see Figure 7. This collection was taken without optimization at this stage, in contrast to Scenario 2. Clusters are detectable in the plot, so a clustering method should be able to find the potential centers for each cluster. Furthermore, with this plot, unstable points and the overlap of some points can be detected.

### 2.3. Data Mining: Subtractive Clustering

**Figure 8:**
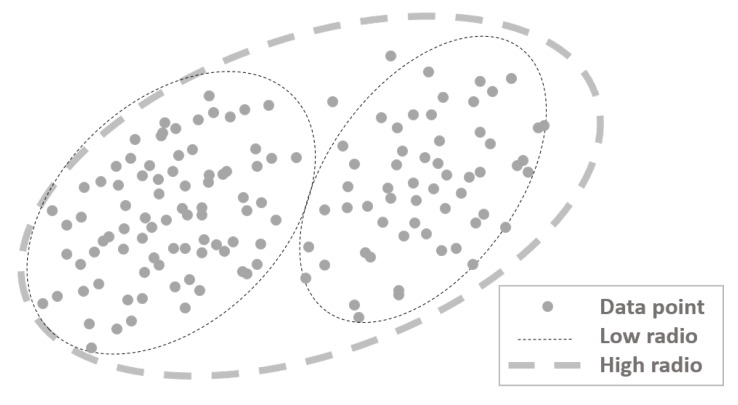
Radius of influence in the subtractive clustering difference.

The approach of data mining is to discover, predict and forecast the possible actions that will occur with some certainty for each prediction [23]. The clustering of numerical data is one type of data mining, and its objective is to identify natural groups or clusters from a big set of data to generate a concise representation of the system behavior [21]. Subtractive clustering was used in this ILM; this algorithm extracts a set of rules that models the data behavior by finding potential centers in the dataset given an influence radius parameter; this is the reason it is a semi-supervised algorithm. The influence radius defines the range of the search for clusters in a dataset; a low radius implies closer data members; a high radius amplifies the range of the search and finds bigger and a lesser number of clusters, as shown in Figure 8. The number of clusters defines the number of rules of the FLS. Centers and the standard deviation of the clusters were used to construct Gaussian membership functions from the antecedents of the fuzzy rules. For a detailed description of the subtractive algorithm, see [24]. Additionally, a linear least squares estimation was used to determine each consequent equation for each rule [19].

One general way to analyze the data behavior is by the distance level between zones, as shown in Table 2. If the collection of data were executed in continuous zones, an overlap between data can occur, so clustering can be complicated. Therefore, the radius for the search of clusters should be lower than 0.4 to get smaller groups with closer data in order to get more rules and a more detailed description of the data behavior. The second level occurs when zones are relatively closer, but not too far; in this case, data can be visualized clearly if a plot representation is possible. The radius of influence in the clustering algorithm is not too low, nor too high (approximately between 0.4 and 0.7). Depending on the number of collected data, the computational cost of clustering will be reduced compared to closer zones. Nevertheless, a high membership grade of more than one zone between a few sets of input data can still exist. The last level based on the distance between zones happens when zones are far away from each other; in this case, the probability that datasets can belong at the same time to different zones is lower, then it is computationally easier to determine the groups in the collected data, as long as the radius of influence is appropriately configured. These three cases and the levels of the radius are approximations based on ideal conditions without interference.

**Table 2:** Approximations of the influence radius levels depending on the distance between zones.

Radius Level	Distance between Zones (Meters)	Radius Value
Low	<5	0.1 to 0.4
Medium	<10	0.4 to 0.7
High	>10	0.7 to 0.9

On the other hand, Table 3 shows a variation of the radius levels considering noise or interference in the collected data determined by the data behavior during experimentation. For null or low levels of noise, the radius level is the same; as mentioned before, it tends to increase as the distance between zones increases. The presence of noise or interference leads to the need to obtain more rules that describe the system in more detail. In this case, it is necessary to reduce the influence radius of the clustering algorithm in order to divide the dataset into more clusters. Then, based on ideal conditions without interference, the influence radius decreases as the noise level increases for every level of distance.

**Table 3:** Approximations of the influence radius levels depending on the distance and noise.

		Noise Level		
		Low	Medium	High
**Distance**	Long	0.7 to 0.9	0.4 to 0.7	0.1 to 0.4
	Medium	0.4 to 0.7	0.1 to 0.4	0.1 to 0.2
	Short	0.1 to 0.4	0.1 to 0.2	0.1

Some features of the clusters, such as the size and their number, were controlled by specific parameters involved in different clustering techniques. Generally speaking, a greater quantity of rules describes the behavior of the system in more detail, so a better approximation of the evaluation or better accuracy can be achieved [25]. This is important considering the computational cost and robustness of the system to define the minimal resources needed to obtain acceptable times for both obtaining the number of clusters and the FLS evaluation. It is noteworthy that in recent years, several techniques for optimizing the fuzzy rules of a system are still being researched to obtain better results, as well as to reduce the number of rules. Although during this experimentation, those techniques were not explored, it can be seen as future optimization work.

With the collected data, it is possible to find the patterns to estimate location. For example, if data come from three different APs and if the data were plotted into 3 axes, as in Figure 1, in perfect circumstances, *n* cluster groups should be visualized, where *n* is the number of zones. In this way, each zone was described for each cluster. The clusters were formed because values have a strong relationship between them. The possible clusters in a set of data depend on the nature of the collected data and the number of data. The number of zones does not always represent the number of groups that give a better system structure.

In order to obtain better results for data mining, it is necessary to obtain enough data to describe each zone. Dispersed data can be obtained when there is noise or interference in Wi-Fi signals during collection. Not enough collected data for each zone is a reason for dispersal, as well. This causes data isolation with a very low or null membership grade for the other clusters, so new clusters will be formed; in the null membership scenario, the unclassified datum will be the center and only member of the cluster.

### 2.4. Type-2 Fuzzy Logic System

Standard logic systems use classic Aristotelian logic with inductive logic or values logic with a set of true concrete values, but human reasoning can be defined more as approximate than precise, because it uses approximate values (for example: true, very true, more or less true, less true, false, not so false, *etc*.), that is why this kind of reasoning logic is called fuzzy logic, where the system solution is approximate [26]. At the same time, in the real world, many problems can be solved with a fuzzy approach considering that variables in the real world have uncertainties. The concept of Type-2 fuzzy logic was introduced by Zadeh [27] as an extension of the usual concept of Type-1 fuzzy sets. A Type-2 fuzzy set is characterized for a membership function where the grade of membership of each element of the universe is a membership function in the range of 0 to 1, in contrast to Type 1, where values of the membership are only numerical values in the range of 0 to 1. A Type-2 fuzzy inference system (FIS) can be used when it is not possible to determine the exact membership grade or when there exist linguistic or numerical uncertainties about the rules [28].

**Figure 9:**
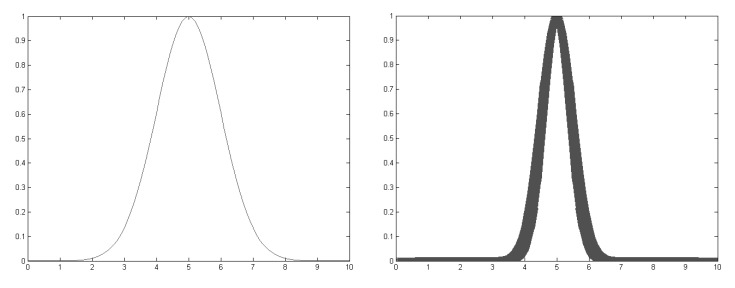
Fuzzy set Type-1 (**left**) and Type-2 (**right**).

Figure 9 shows a Type-1 fuzzy set on the left and Type-2 on the right, represented as a Gaussian membership function. The obtained clusters from the data mining process obtain the parameters needed to almost directly create a Type-1 Gaussian membership function that conforms to the fuzzy rules from the FIS for the proposed ILM. These parameters are the center *c* and standard deviation *σ* from Equation (1). A Type-2 Gaussian membership function has an uncertainty parameter for modeling it in the width or the standard deviation.

(1) $$\mu \left(x_{1}\right)=exp\left[-\frac{{\left(x_{1}-c_{1}\right)}^{2}}{2\sigma_{1}^{2}}\right]$$

**Figure 10:**
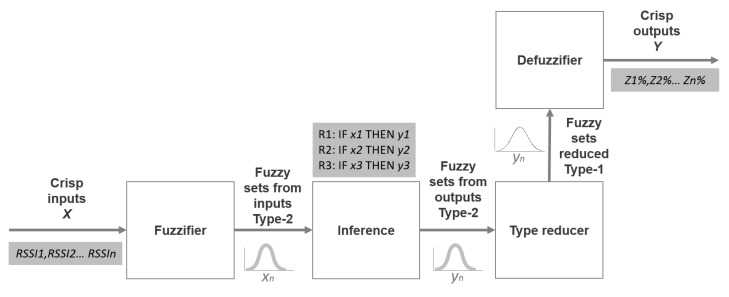
Type-2 fuzzy inference system (FIS) structure.

A Type-2 FIS consists of 4 parts: fuzzifier, knowledge base or rules, fuzzy inference machine and output processor, as Figure 10 shows. A reduction of the type is necessary in the output processor to convert a dataset from Type-2 to Type-1 [29].

**Figure 11:**
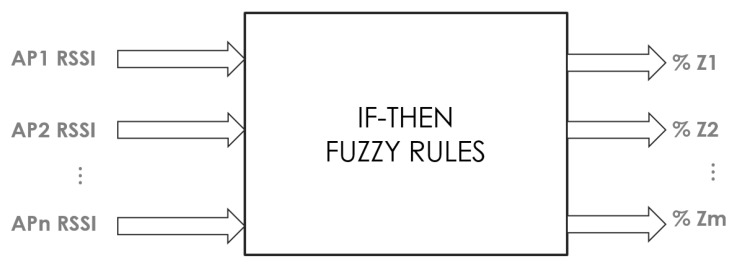
FIS mapping inputs to outputs for ILM.

The quantity of access points used to obtain the RSSI data defines the number of inputs of the FIS; meanwhile, the quantity of zones where location are to be estimated defines the number of outputs. During the online phase, the FIS receives one RSSI per input, and the output is the membership grade of the entire input set regarding each output or zone; see Figure 11. This approach makes it possible to discover the location of the device between zones when one set of inputs gives an output with two zones with the same membership grade, reducing the fingerprinting work. For this ILM, a Takagi–Sugeno–Kang (TSK) FIS was used because of the simplicity of the consequent part with an equation system, so that the evaluation time of the system was reduced.

**Figure 12:**
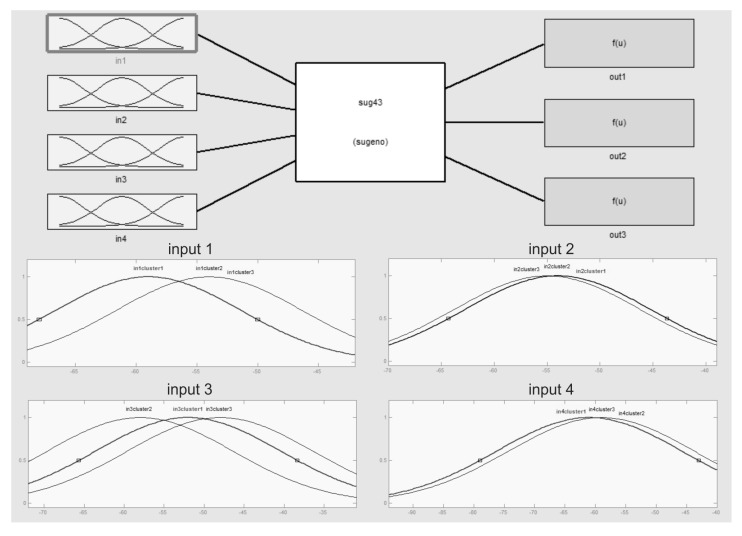
TSK FIS structure with four inputs, three rules and three outputs.

Figure 12 is a TSK Type-1 FIS structure constructed with collected data in Scenario 2 using the MATLAB fuzzy tool. The sample FIS has four fuzzy sets as inputs (RSSIs), three IF-THEN rules and three outputs (zones) with linear equations. It is similar to that generated with the Java tool implemented in the mobile device, because even if we use Type-2 fuzzy logic, RSSI uncertainty was not yet modeled and implemented on the input Gaussian membership functions; therefore, these are like Type-1 input functions with implicit uncertainty, as the four extended inputs in the figure show. The Type-2 tool used in the proposed ILM reduces to the Type 1 used during the evaluation stage.

## 3. Results

The results were generated with the proposed ILM and using collected data from two scenarios, one with three APs and three zones that were not so close to each other; and the other with four APs and three closer zones; also, this last scenario implemented the faulty AP signal remover Algorithm 1.

### 3.1. Scenario 1

Table 4 shows the results using subtractive clustering and Takagi–Sugeno FIS for the localization in Scenario 1. A total of 1680 samples per AP were taken. In this case, the modified parameter was the influence radius or granularity grade from 0.9 to 0.1. Because the distance between each zone is not short, the influence radius from 0.1 to 0.7 has acceptable results. A low radius tends to generate more clusters, because it tries to cluster data that are closer to each other based on a low radius of search. A high radius should be used with clusters with dispersed data or low density, so generally, it will obtain a smaller number of clusters. Subtractive clusters are more optimal than clusters found by fuzzy c-means in this case study, as you can see in the comparison work [18]. Note that the data et was obtained in zones that were not so close; therefore, the descriptive clusters of the system do not represent an expensive computational search.

The regression coefficient estimates the relationship between the evaluation results and the target values. For this table, the evaluation results are obtained by evaluating the constructed system with original inputs; target values are the original outputs used to build the system. Results should match the best with target values (more than 75%; measurements variations can be handled by sampling); if not, this means that the system has not learned the rules well. The accuracy of the proposed method changes the influence radius on the subtractive clustering, because it defines the rules of the system. It is noteworthy that no optimization rule was implemented, so it is possible to reduce the number of rules in the system once the clustering is done. Almost every radius gives acceptable results. In the next section, another validation method of the model is discussed in detail.

**Table 4:** Comparison table of the Takagi–Sugeno method with three inputs and three outputs.

Radius	Clusters	Generation Time (s)	Evaluation Time (ms)	Regression Coefficient
0.9	4	217.96	3.1–3.8	0.7361
0.7	4	217.91	3.1 to 5.2	0.9143
0.5	4	220.90	3.3 to 5.5	0.9245
0.4	4	224.36	4.2 to 5.1	0.9307
0.3	4	235.08	3.1 to 4.0	0.9345
0.2	8	222.05	7.0 to 10.0	0.9528
0.1	19	242.30	12.4–17.8	0.9741

### 3.2. Scenario 2

On the other hand, Table 5 is a sample of Scenario 2 using four APs instead of three for three closer zones. In this case, a total of 1224 for each AP sample was taken.

**Table 5:** Sample of collected data for Scenario 2.

Zone	Input 1	Input 2	Input 3	Input 4	Output 1	Output 2	Output 3
1	−53	−50	−56	−57	1	0	0
1	−46	−57	−60	−52	1	0	0
1	−48	−61	−47	−56	1	0	0
2	−55	−53	−58	−56	0	1	0
2	−56	−45	−53	−94	0	1	0
2	−45	−57	−61	−61	0	1	0
3	−60	−57	−47	−57	0	0	1
3	−54	−60	−56	−58	0	0	1
3	−51	−54	−47	−75	0	0	1
…	…	…	…	…	…	…	

Table 6 shows the results using the subtractive algorithm to find the FIS rules on the mobile device with the Android application. The S0 dataset was used to compare the results representing one of many datasets that did not give a good estimation coefficient with closer zones. S0 was a dataset of 1344 data per three inputs (APs) and four outputs (number of zones). Even in very close zones, it is possible to have good accuracy, as the regression coefficient from S2 indicates, with a low influence radius (less than 0.4). A good estimation was not able to be executed in a mobile device in very close zones, as the results of S0 show. In contrast, an acceptable estimation with an influence radius of 0.4 in S2 with the remover Algorithm 1 is enough. The elapsed time during clustering and FIS construction was between 150 to 500 s, with one exception where the clustering of the dataset from S1 was not possible to complete only by using the MATLAB tool. Furthermore, an increment of the number of rules in S2 compared to S0 was explained because of the addition of one input.

**Table 6:** Comparison table of Takagi–Sugeno FISs.

Radius	Clusters S0	Regression Coeff. S0	Clusters S2	Regression Coeff. S2
0.9	4	0.5237	3	0.5197
0.7	4	0.5219	4	0.6036
0.5	4	0.5173	8	0.7835
0.4	6	0.5343	13	0.909
0.3	12	0.5874	35	0.9993
0.2	31	0.7475	49	0.9993
0.1	-	-	62	0.9993

Note that from a 0.4 to a 0.1 radius (13 to 62 fuzzy rules), the accuracy in this case did not increase or decrease dramatically. Therefore, it is not necessary to force the device to compute a search with a lower radius. The goal is to find the most acceptable accuracy with less computational cost.

### 3.3. Method Evaluation

The evaluation of this case study was made in the post-online stage with different datasets using the MALTAB software tool. For the evaluation of the proposed ILM, two methods were used. First, a linear regression with the purpose of comparing and estimating if the system works with the same data on which the system was developed was performed. Furthermore, a test was done to see if the FIS can provide an effective evaluation of at least 80% of the data. The results of these methods are shown in Table 5 and Table 6. Again, an exhaustive evaluation method for the validation of the models based on data mining called 5 × 2 cross-validation was used. The ILM proposed uses data mining techniques. According to the evidence or the set of collected data, a knowledge base is formed; in this case, the TSK fuzzy inference system. To evaluate the quality of the formed model, different techniques exist. Most of them consist of separating and permuting the evaluation data and the training data to have greater independence between them, avoiding the over-adjusting of the models (giving good results only with this set of data) that does not generalize to other data [30]. Algorithm 2 is a typical validation of the models.

**Figure 13:**
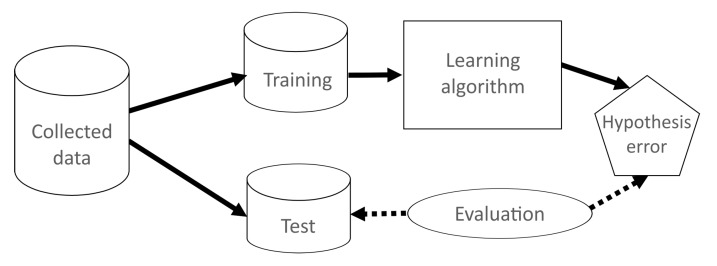
Evaluation of the data mining methods’ process.

Figure 13 shows the general process of the correct evaluation of the model. The original data are divided into two type of sets, training and test data. Training data generate the model with a learning algorithm, subtractive clustering in this case. Once the model is obtained, the test dataset is used to evaluate the model. Furthermore, a confusion matrix is used to evaluate the model to detect how many data are correctly classified. Then, a hypothesis error is calculated. The number of iterations and errors to calculate the final average depends on the technique used.

A confusion matrix sample is shown in Table 7. This sample is from Scenario 1 and shows three different categories or output classes vertically and three different targets horizontally. Output is the result of FIS evaluation. Target classes represent the objective or expected values horizontally. Then, 267 are true positives of the Output 1 and Target 1; 261 of Output 1 and Target 2; and 260 of Output 3 and Target 3. Therefore, a total of 93.8% of the data match, and the confusion error is 6.2%. The error percentage obtained by the difference of 100 and the percentage of classified data.

**Table 7:** Sample of a confusion matrix from ILM.

	Target 1	Target 2	Target 3	% Classified	% Error
Output 1	**267**	2	8	96.4	3.6
Output 2	31	**261**	8	87.0	13.0
Output 3	0	3	**260**	98.9	1.1
% Classified	89.6	98.1	94.2	**93.8**	
% Error	10.4	1.9	5.8		**6.2**

The original cross-validation uses *k* = 10, which divides the set of data into 10 parts sharing 80% of the data in each iteration; this overlap of the data can affect the estimation quality. For better independence between data, a 5 × 2 cross-validation was used, this method realizes five iterations with *k* = 2; in each iteration, data are randomly re-ordered; at the same time, in each iteration, data are divided into two sets (training and test); the error is obtained, and then, the sets are exchanged to obtain the error again. At the end, the final error is obtained by the average. An acceptable final error is less than 25%. If the error is higher, then the influence radius of the subtractive clustering should be adjusted. In case modifying the influence radius does not enable a correct description of the system, an expert can modify the rules to achieve the desired results; in the case of a lack of data, enough data to describe each zone need to be collected; in the worst scenario, data are corrupted or an abnormality happens during the last collection, and the process should re-start with a new collection of data.

**Algorithm 2** Simple cross-validation algorithm.**Require:** DataSet is the collected data set and defines *k* value1:divide the evidence DataSet into *k* subsets in equal sizes2:**for** *k* to *k*=0 **do**3:  a hypothesis is learned for the union of *k*-1 subsets4:  remaining set is used to calculate a partial error of the sample5:  reduce *k*-16:**end** **for**7:finalerror is the average of the experiments with *k* subsets8:**return** finalerror

Table 8 shows the total percentage error averages using collected data from Scenario 1 and Scenario 2. There were problems during the location of closer zones with a very low rate of accuracy in the method; implementing an algorithm to remove faulty signals and using four APs solved the problem. The Scenario 2 dataset was obtained with the optimization algorithm. TSK An and TSK Bn are the hypothesis errors of the two iterations for each *k*. TSK Avgs is the average of TSK An and TSK Bn. The final error is the average of TSK Avgs. Better accuracy was obtained with the optimized method, as the final error from Scenario 2 indicates. TSK from S1 has eight rules and corresponds to an influence radius of 0.5. TSK from S2 has 13 rules and corresponds to a radius of 0.4. This is an example of the hypothesis error reduction.

**Table 8:** Average of the percentage of hypothesis errors from the S1 and S2 datasets.

k	APs	Radius	Rules	TSK A1	TSK B1	TSK Avgs 1	APs	Radius	Rules	TSK A2	TSK B2	TSK Avgs 2
1	3	0.5	8	6.2	6.1	6.15	4	0.4	13	3.5	3.7	3.6
2				6.7	6.3	6.5				3.7	3.8	3.75
3				7.4	6.2	6.8				3.8	4.3	4.1
4				6.9	5.4	6.15				3.3	3.1	3.2
5				7.3	5.6	6.25				3.9	3.9	3.9
Final errors						**6.37**						**3.71**

### 3.4. Power Consumption

The experimental process was performed with a Samsung Galaxy Tab 4 7.0 with a 3.8-, 4-KmAh lithium ion battery. The usage estimations were obtained using a power monitor for Android-based mobile applications called PowerTutor, which implements a power model construction technique; more details about the model and the application are in [31].

Table 9 shows the average of power consumption during the collection of the datasets of 100, 1000 and 10,000 samples. The average temperature of the battery was about 26 and 28 ${}^{{}^{\circ}}$C. The collection time (indicated in seconds) varies depending on the quantity of samples needed and the environment conditions, like noise, because there are mobile entities or many devices in the same band or channel that alter the availability of the APs. The average power consumption was 75.6524 mW. Finally, the total power consumption used was about 81.0647 mW for 100 samples, 74.1559 mW for 1000 and 71.7367 mW for 10,000 samples.

**Table 9:** Battery consumption of the implemented method per sample.

Number of Samples	Temperature (°C)	Collection Time (s)	Average Consumption (mW/h)
100	28	282	81.0647
1000	28	2112	74.1559
10,000	26	25,280	71.7367
			**75.6524**

**Figure 14:**
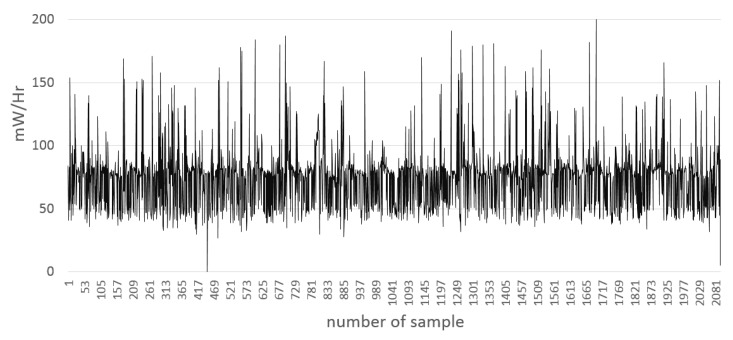
Example of the power consumption chart.

The following Figure 14 has the behavior of the power consumption during the elapsed time for a collection of 1000 RSSI samples. The behavior is similar to the other collections from 5 to 200 mW, approximately. The vertical axis represents the power consumption in milliwatts; meanwhile, the horizontal axis represents the number of power samples from the PowerTutor tool during the elapsed time (35 min, as indicated in Table 9).

Moreover, Table 10 shows the power consumption results by the data mining method used to generate the fuzzy inference rules by finding clusters in the collected data. The first column shows the number of samples per input and output, the number of rules generated by the subtractive clustering algorithm, the elapsed time to obtain the clusters in seconds and the power consumption average in milliwatts. The battery temperature was 26 ${}^{{}^{\circ}}$C.

**Table 10:** Battery consumption of the implemented method per generated cluster or rule.

Number of Samples	Number of Rules	Generation Time (s)	Avg. Consumption (mW/h)
1224	3	121.2110	332.25
1681	4	226.0839	334.99
1681	8	215.4916	345.09
1224	50	301.8800	339.05

**Figure 15:**
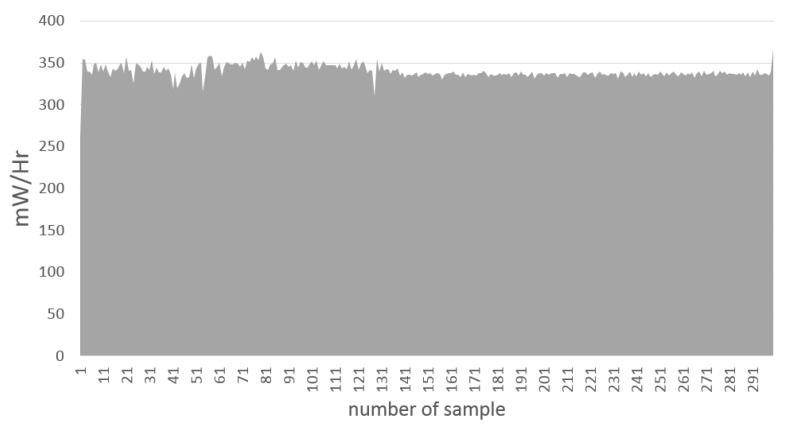
Example of power consumption area chart.

Figure 15 is an area chart with the power consumption values obtained with PowerTutor during the data mining process to obtain 50 rules from a dataset of 1224 samples. The average in this case was about 339.05 mW, and the elapsed time was 301.88 s, as indicated in Table 10.

Table 11 shows the number of inputs and outputs of the FIS used, the number of rules, the elapsed time during the evaluation of the FIS average in milliseconds and the power consumption of the battery in milliwatts units. The battery temperature was between 23 and 24 ${}^{{}^{\circ}}$C. The obtained results are the consumption averages, which include an interface action based on the resulting estimation from the FIS. As can be seen, the major consumption of power is at the collection stage, because of the amount of time involved, but in some cases, the search for clusters can be slow and expensive, as well, mainly because the power cost of the data mining operations is higher than the collection instructions. Both stages of the process usually are developed by experts that train the systems, so the final user should not be concerned about power consumption or time costs; usually, final users only want to be located, which is part of the final stage FIS evaluation.

**Table 11:** Battery consumption of the implemented method per the evaluation of rules (estimation of location).

Number of Inputs	Number of Outputs	Number of Rules	FIS Eval. Time (ms)	Avg. Consumption (mW/h)
4	3	3	6.6528	21.31
4	3	7	18.34	38.77
4	3	35	21.79	70.11
4	3	62	75.50	178.8

**Figure 16:**
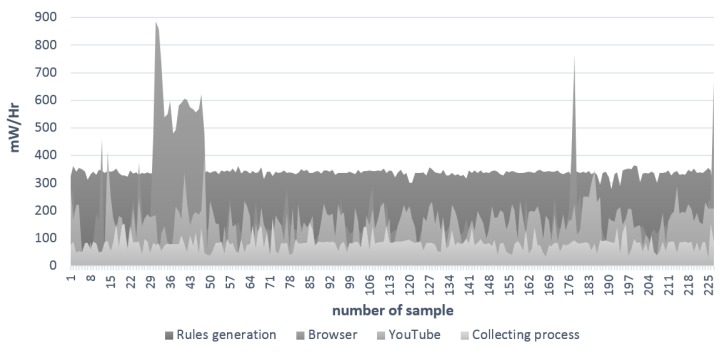
Comparison chart between different processes’ consumption.

In order to get a better view of the consumption, Figure 16 shows a comparison chart between the average power consumption of the mobile web browser, YouTube application and two processes of the proposed method: the collection of data and the generation of rules. As can be seen, the process with less average consumption (per h estimation) is the collecting process with 81.3 mW; in practice, this is the most expensive, because it requires the longest execution time. The YouTube application has an average of 161.56 mW, similar to the use of the browser with 174.2 mW; in both applications, the consumption depends on the content to load, and normally, it is unstable, with spikes in the consumption measures. The high average estimation per hour is for the generation of the rules process, with 338.3 mW, as mentioned in the description of Table 10; but in practice, it depends on how long it takes to find the clusters in the obtained dataset.

### 3.5. Discussion

Previous experimentation was developed in order to test the method in different indoor spaces. This section describes and compares in detail three different case studies; one in the interactive museum shown in Section 2.2.3 as Case Study 1; one between classrooms of a university building as Case Study 2; and one between different rooms of a house as Case Study 3.

#### 3.5.1. Case Study 2

**Figure 17:**
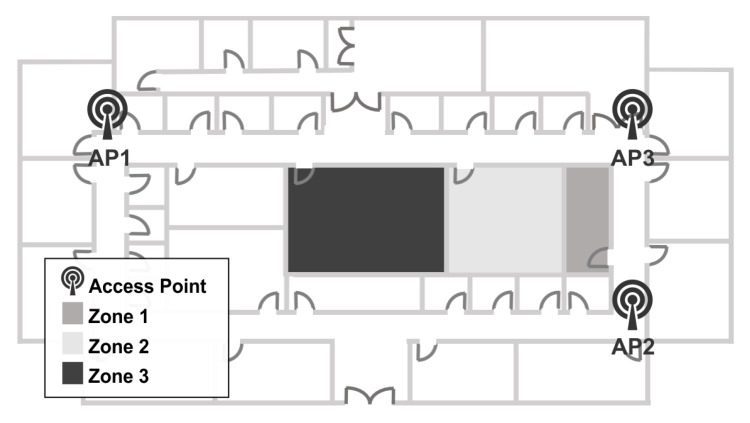
Computer science floor, Case Study 2.

Figure 17 shows three zones from the university floor from the data of Case Study 2. Zone 1 is a complex systems laboratory; Zone 2 is a projects classroom; and Zone 3 is technical support. The access points used are located in three corners of the building and are represented as AP1, AP2 and AP3; they are Cisco Aironets 2700 Series Access Points AIR-CAP2702I-x-K9. Because it was an engineering floor, there were at least 15 other APs, many connected devices and people in motion, that resulted in noisy conditions; this is handled by the implemented algorithm that filters faulty measurements, but this extends the normal collection time. The testing device during collection, FIS generation and evaluation was a Samsung Galaxy Tab 4 7.0, 1.4-Hz Quad Core Processor with a 4-KmAh battery.

Because of the noisy conditions and the short distance between zones, based on Table 2, a radius of 0.1 was selected in order to obtain optimal results. Table 12 shows a sample of the results during the evaluation of the obtained FIS. The zone column indicate the zone where the sample was taken; the RSSI columns indicate the samples of scanned values from the three APs selected; and the last zone columns indicate the evaluation results of the location FIS generated before. A more positive value indicates greater compatibility of the sample with the zone. For example, from the first sample in Zone 1, the FIS output of the evaluation was 93.24% belonging to Zone 1, −4.7% membership to one 2 and 11.45% membership to Zone 3; therefore, the sample from the first row belongs to Zone 1.

**Table 12:** Sample of the location evaluation, Case Study 2.

Zone	RSSI AP1	RSSI AP2	RSSI AP3	Zone 1 %	Zone 2 %	Zone 3 %
1	−55	−61	−79	0.9324	−0.0470	0.1146
1	−50	−67	−90	0.9769	0.0847	−0.0616
1	−55	−68	−77	1.0916	−0.0204	−0.0712
2	−73	−61	−58	−11.162	6.9250	5.2370
2	−66	−69	−51	0.7123	0.2884	−7.76 × 10${}^{-4}$
2	−53	−75	−71	−0.0036	1.0495	−0.0458
3	−67	−71	−78	0.3911	−0.6156	1.2245
3	−70	−72	−76	−0.0397	0.0422	0.9975
3	−65	−71	−72	1.3104	−1.1420	0.8316

#### 3.5.2. Case Study 3

**Figure 18:**
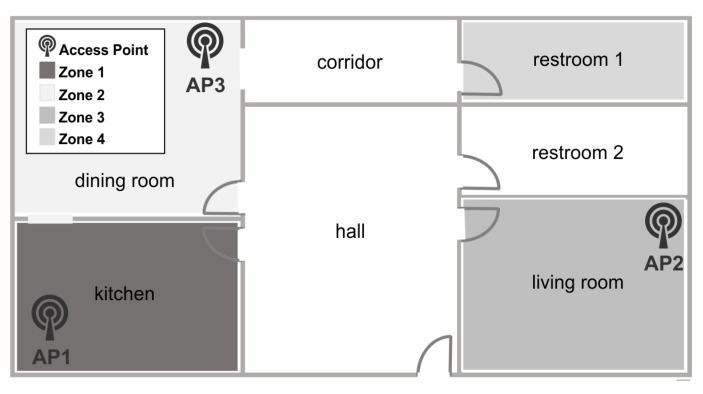
House, first floor, Case Study 3.

In the medical field, for example, there is interest in indoor location monitoring of patients to find patterns or for healthcare situations in their homes. This case study can be applied to this kind of scenario. Figure 18 shows four zones representing different rooms of a house: Zone 1 is the kitchen; Zone 2 is the dining room; Zone 3 is the living room; and Zone 4 is the restroom. Access points are positioned and represented as AP1, AP2 and AP3. AP1 is a HUAWEI Router model HG532e 2.4 GHz; AP2 is a Belkin Wireless G Router 2.4 GHz 802.11g Model No. F5D7230-40; and AP3 is an Air Port Extreme by Apple Model No. A1034. In this case, only two APs and a few basic domestic devices were connected and near the space, so, in contrast to Case 2, this case has normal and low interference conditions, so a very short time of collection. The testing device during collection, FIS generation and evaluation was a Samsung Galaxy Tab 4 7.0, 1.4-Hz Quad Core Processor with a 4-KmAh battery.

Because of the stable conditions of this space, using a radius from 0.9 to 0.1 during data mining results in an effectiveness of 93.4% to 99.7%, respectively. In order to be able to compare to other case studies and to obtain the best effectiveness results, the selected radius was 0.1. Table 13 shows a sample of the location tests using the generated FIS with the collected data. The zone column indicates the zone from the collected sample; the RSSIs columns indicate the scanned signals from each AP (inputs); and the zones’ columns indicate the output resulting from the evaluation of the location FIS with the sample inputs. Each output indicates the membership grade of the inputs with each zone; for example, in the first row, the first sample was taken in Zone 1; the inputs were −56, −51 and −83 from AP1, AP2 and AP3, respectively; then, the membership grades of this sample with each zone are 103.4%, 0.9%, −1.77% and −3.07% for Zones 1, 2, 3 and 4, respectively. Therefore, the system indicates with 103.4% of certainty that the user is in Zone 1.

**Table 13:** Sample of location evaluation, Case Study 3.

Zone	RSSI AP1	RSSI AP2	RSSI AP3	Zone 1 %	Zone 2 %	Zone 3 %	Zone 4 %
1	−56	−51	−83	1.0393	0.0090	−0.0177	−0.0307
1	−61	−88	−44	1.0828	−0.0025	−0.0802	−6.57 × 10${}^{-5}$
2	−33	−95	−57	0.0042	1.0019	−0.0054	−6.63 × 10${}^{-4}$
2	−39	−75	−92	−1.5252	2.9223	−0.0013	−0.3958
3	−64	−53	−60	−0.0013	0.0048	0.9942	0.0023
3	−65	−59	−68	0.0025	−0.0112	0.8886	0.1202
4	−57	−85	−71	0.1235	−0.1052	−0.0013	0.9830
4	−62	−88	−90	0.0079	0.0031	−3.8366 × 10${}^{-4}$	0.9894

#### 3.5.3. Comparison of Case Studies

Table 14 shows the percentages of the effectiveness based on the evaluation of the location FIS generated with the collected dataset in each case study. Case Study 1 S1 and S2 were described in Section 2.2.3 and were developed in an interactive museum with two scenarios with variations in the distances between zones. Meanwhile, Case Study 2 was developed in a university building of science computing with high levels of interference and Case Study 3 in a house with low levels of interference. A case study with separate zones was proposed, developed and discussed in [16]. This table shows that it is possible to estimate indoor location with more than 94% effectiveness using the proposed method; better accuracy can be achieved with supplementary techniques in the offline or online stage (as statistical sampling).

**Table 14:** Comparison table of three different case studies.

Case Study	Number of APs	Number of Zones	Influence Radius	Number of Rules	Gen. Time (s)	Effectiveness
1 S1	3	3	0.1	19	242.30	0.999
1 S2	4	3	0.1	62	504.23	0.974
2	3	3	0.1	29	44.56	0.943
3	3	4	0.1	54	274.61	0.997

Another aspect to highlight is that when increasing the number of inputs or outputs, the complexity of the system increases, then the data mining process extends in time; even so, it is possible to generate FIS rules with low estimation error, as shown in table, by knowing the space conditions and selecting the corresponding radius for clustering. Finally, the generation time also is altered by data patterns depending on the noise and distance between zones.

## 4. Conclusions

This alternative hybrid intelligent method estimates the indoor location of mobile devices by zones using RSSIs from selected APs successfully. This different approach indicates in which zone a user or a device is, instead of exact coordinates. It is an alternative proposition from the existing works and was tested on a mobile device from the offline to the online stage. This is a novel application of the data mining technique of subtractive clustering with a fuzzy logic approach, which opens the possibility of modeling the real uncertainties of Wi-Fi signals. An important advantage of this technique is that it is semi-supervised, so reducing the the human work during the process after collecting the data is significant. Future work is developing an algorithm to obtain the uncertainty from RSSIs to add it to the FIS inputs.

While testing the clustering method on several datasets, it was checked that a higher influence radius on clustering produces fewer clusters, therefore fewer rules; a lower radius produces more clusters; then, more rules that describe in more detail the system behavior, achieving a better accuracy. At the same time, better accuracy has the cost of time and increases the number of rules. During this work, comparisons of resulting FISs are shown. In the online stage, the time response of the evaluation of the location system with a new input set is immediate, because of the TSK systems’s structure.

Power consumption tests showed that the final user location is estimated online with a short time of response and lower power consumption, which is increased by the complexity of the FIS determined with a certain number of inputs, outputs and rules. The higher consumption is in the collection of samples, because of the amount of time it takes to complete them. Meanwhile, the subtractive algorithm has a medium cost depending on the complexity of finding the clusters in a dataset. It should be noted that in order to reduced the cost of time (and then, power costs), programmers can implements strategies in three phases, collecting, the generation of rules and the estimation of the location, depending on the purposes of the final application.

A limitation of this method, due to the use of radius mapping, is that conditions may vary the base model; then, a differential or weighting approach instead of raw RSSIs can be used in the offline stage to mitigate the impact of these variations in the generated model, making it more adaptable; additionally, filters in the collection stage can be implemented to remove the peaks and drops of the signals. If variations are not radical, model profiles can be handled to use the corresponding FIS adapted to certain environment circumstances. The experimentation with different scenarios gives us more future challenges.

The Android tools used to implement the ILM proposed were developed as past work to validate the method. At the same time, validation techniques of data mining were used in the post-online stage, giving acceptable results in near and more separated zones, as well. The developed method and tools can be used in mobile indoor location-based applications. This work discussed the advantages of Wi-Fi technology over others and the importance of location as a fundamental element to know the context, to contribute to the development of devices with situational awareness.
